# Coil-assisted retrograde transvenous obliteration for managing complex gastric variceal bleeding: a pediatric case report and review of techniques

**DOI:** 10.3389/fped.2025.1558097

**Published:** 2025-04-24

**Authors:** Amirhossein Hosseini, Iman Kiani, Mitra Khalili, Mina Alibeik, Arash Khameneh Bagheri

**Affiliations:** ^1^ Pediatric Gastroenterology, Hepatology and Nutrition Research Center, Research Institute for Children’s Health, Shahid Beheshti University of Medical Sciences, Tehran, Iran; ^2^ Students’ Scientific Research Center, Tehran University of Medical Sciences, Tehran, Iran; ^3^ Mofid Children’s Hospital, Shahid Beheshti University of Medical Sciences, Tehran, Iran; ^4^ Department of Pediatric Gastroenterology, School of Medicine, Shahid Beheshti University of Medical Sciences, Tehran, Iran; ^5^ Radiology Department, Shohadaye Tajrish Hospital, Shahid Beheshti University of Medical Sciences, Tehran, Iran

**Keywords:** gastric varices, CARTO, sclerotherapy, case report, endovascular

## Abstract

**Introduction:**

Gastric variceal bleeding is a life-threatening complication of portal hypertension, associated with high morbidity and mortality. While conventional treatments such as endoscopic interventions, pharmacological therapy, and transjugular intrahepatic portosystemic shunt (TIPS) are standard, alternative approaches are needed for high-risk or anatomically complex cases. Coil-Assisted Retrograde Transvenous Obliteration (CARTO) has emerged as a promising technique, combining mechanical and chemical approaches to achieve durable hemostasis.

**Case presentation:**

A 10-year-old girl with a history of congenital spherocytosis and splenectomy presented with hematemesis and a hemoglobin level of 4.4 mg/dl. Initial endoscopy revealed no esophageal varices, but gastric visualization was inconclusive due to active bleeding. CT angiography demonstrated extensive gastric fundal varices, confirmed by transhepatic portography. Management involved CARTO, utilizing sodium tetradecyl sulfate sclerotherapy and coil embolization. Post-procedure imaging showed successful obliteration of varices, and the patient's hemoglobin levels normalized. Follow-up at six months revealed complete symptom resolution.

**Discussion:**

CARTO offers a viable alternative for managing gastric varices in complex cases, particularly when TIPS or BRTO are unsuitable. Compared to BRTO, CARTO is less time-consuming and avoids large sheaths, reducing procedural risks. However, it is technically demanding and cost-intensive, requiring careful patient selection. This case demonstrates CARTO’s effectiveness in achieving hemostasis and managing challenging variceal anatomies.

**Conclusion:**

CARTO is an effective option for managing high-risk gastric varices. Future studies should refine procedural techniques, improve patient selection, and explore advanced embolization materials to optimize outcomes.

## Introduction

Gastric variceal bleeding is a life-threatening complication of portal hypertension, characterized by significant morbidity and mortality rates (1). Unlike esophageal varices, gastric varices are less common but are associated with a higher risk of severe bleeding due to their larger size and higher blood flow (2). Managing gastric variceal hemorrhage remains a clinical challenge, often requiring a multidisciplinary approach. Conventional therapeutic options include endoscopic interventions, pharmacological therapy, and transjugular intrahepatic portosystemic shunt (TIPS) (3). However, in cases where these methods are either ineffective or contraindicated, alternative treatments become necessary.

Coil-assisted sclerotherapy has emerged as a promising technique for managing complex variceal bleeding, particularly in patients with anatomical or technical challenges that preclude other interventions (4). This minimally invasive procedure combines mechanical occlusion with chemical ablation to achieve durable hemostasis and prevent recurrence. By targeting the variceal drainage pathways and obliterating the varices, coil-assisted sclerotherapy offers a viable option for patients with severe bleeding who are unresponsive to standard therapies (5).

In this report, we present the case of a patient with severe gastric bleeding secondary to large gastric varices, successfully treated with coil-assisted sclerotherapy. We detail the diagnostic challenges, procedural approach, and clinical outcomes, stressing the role of this innovative technique in managing high-risk cases of gastric variceal bleeding. This report was in adherence with CAse Report (CARE) guideline (Supplementary Appendix).

## Case presentation

We present the case of a 10-year-old female with a known history of congenital spherocytosis who had undergone splenectomy. She was referred to our center with a complaint of hematemesis, and initial laboratory investigations revealed a hemoglobin level of 4.4 mg/dl. An initial endoscopy revealed no esophageal varices, but visualization of the stomach was inconclusive due to a significant amount of intragastric blood. Despite multiple blood transfusions, her hemoglobin levels showed no improvement.

**Figure 1:**
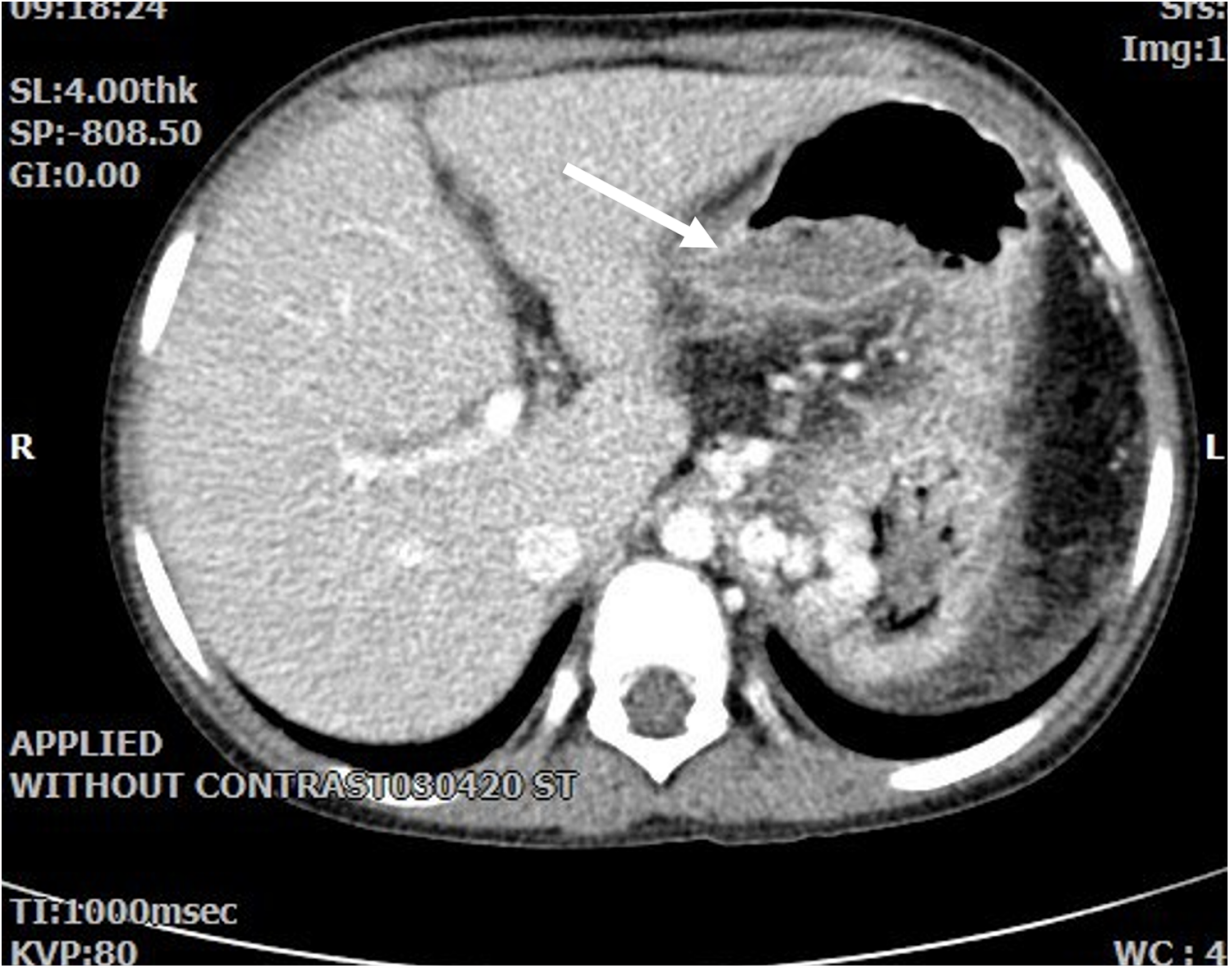
Axial abdominal CT with contrast show evidence of previous splenectomy and extensive gastric varices. Dilated varicose veins protrude intraluminally in fundus with a large gastric intraluminal clot related to recent hemorrhage.

A repeat endoscopy was performed two days later, which confirmed the absence of gastric varices. In abdominal ultrasound, evidence of perigastric collateral vessels was noted, alongside with decreased velocity of portal vein (9 cm/s). To further investigate, a CT scan with an angiography protocol was conducted, which revealed presence of extenstive dilated varices in the fundus of stomach (Figure 1). These varices were actively bleeding at the time the CT.

**Figure 2:**
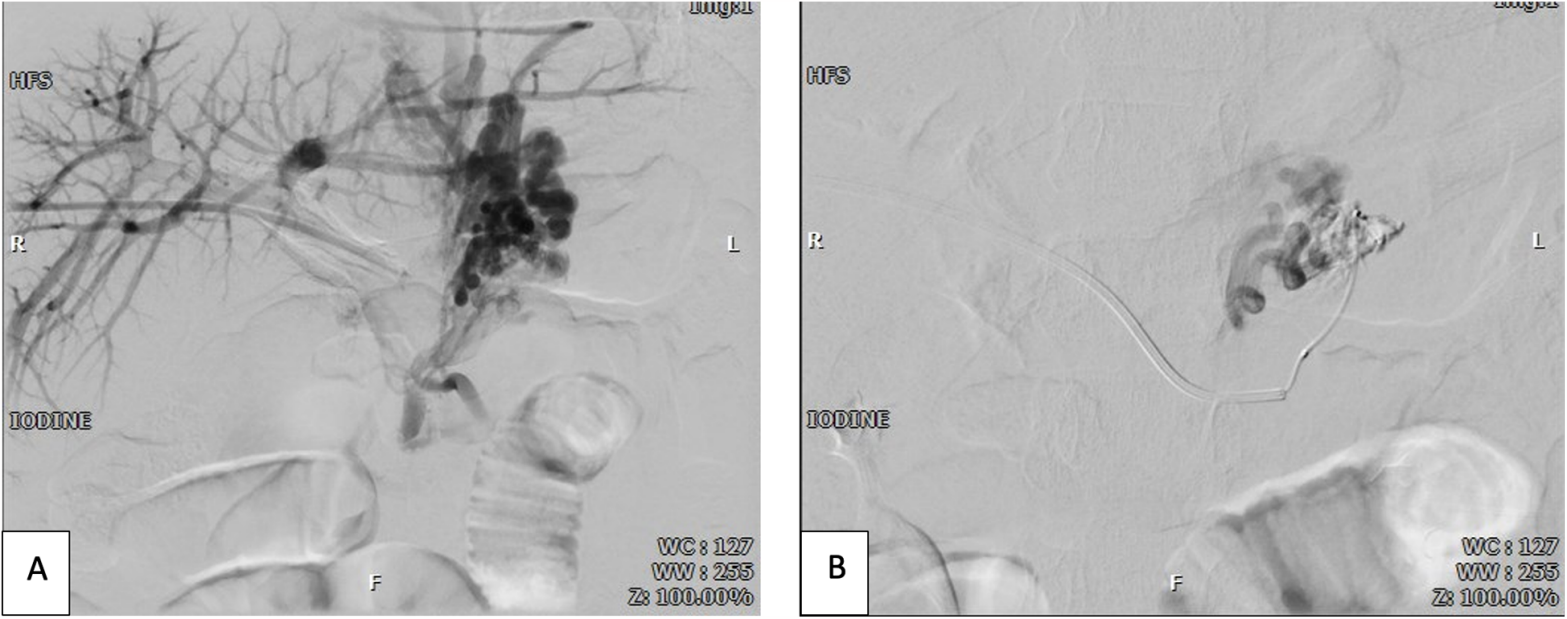
**(A)** Pre-sclerotherapy and pre-coiling transhepatic portography shows dilated varicose veins; **(B)** superselective venography of varicose veins via microcatheter shows porto-systemic shunts.

**Figure 3:**
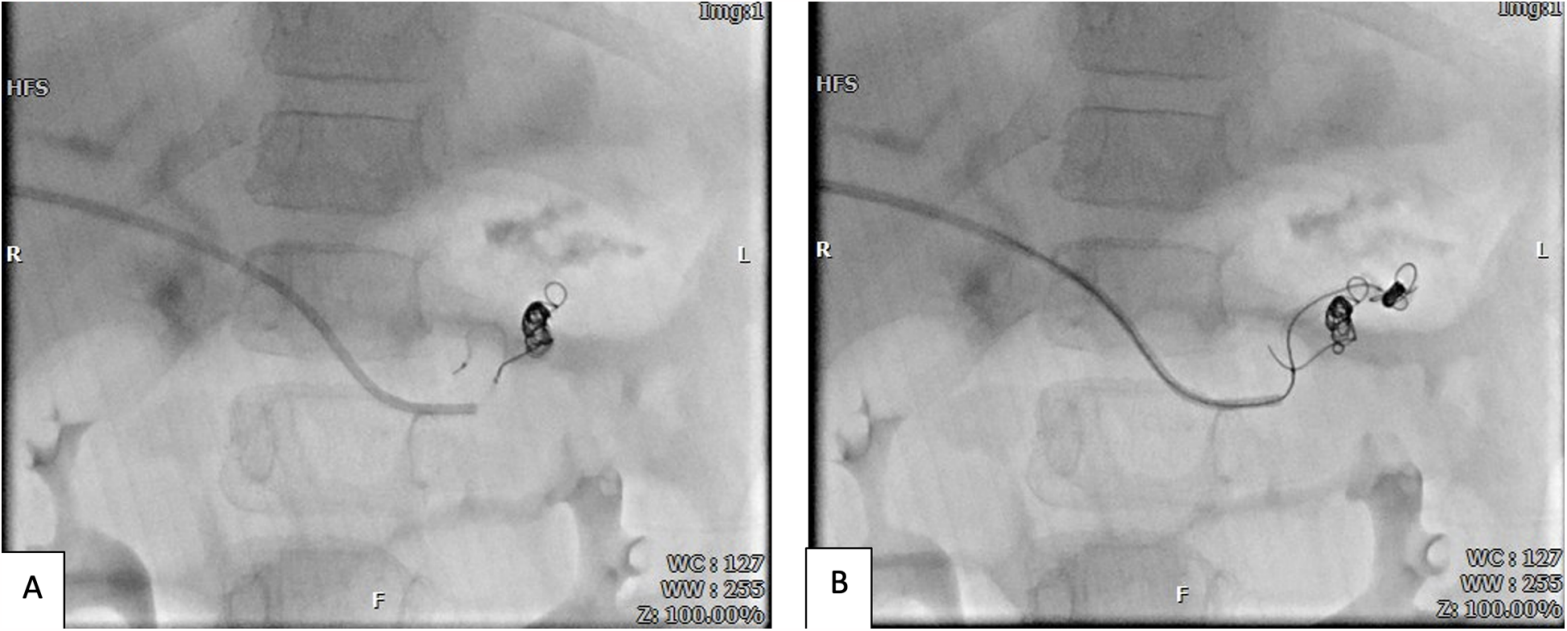
Coiling and sclerotheraphy of the first **(A)** and second **(B)** varicose veins by coils and sodium tetradecyl sulfate (STS) injection via microcatheter.

**Figure 4:**
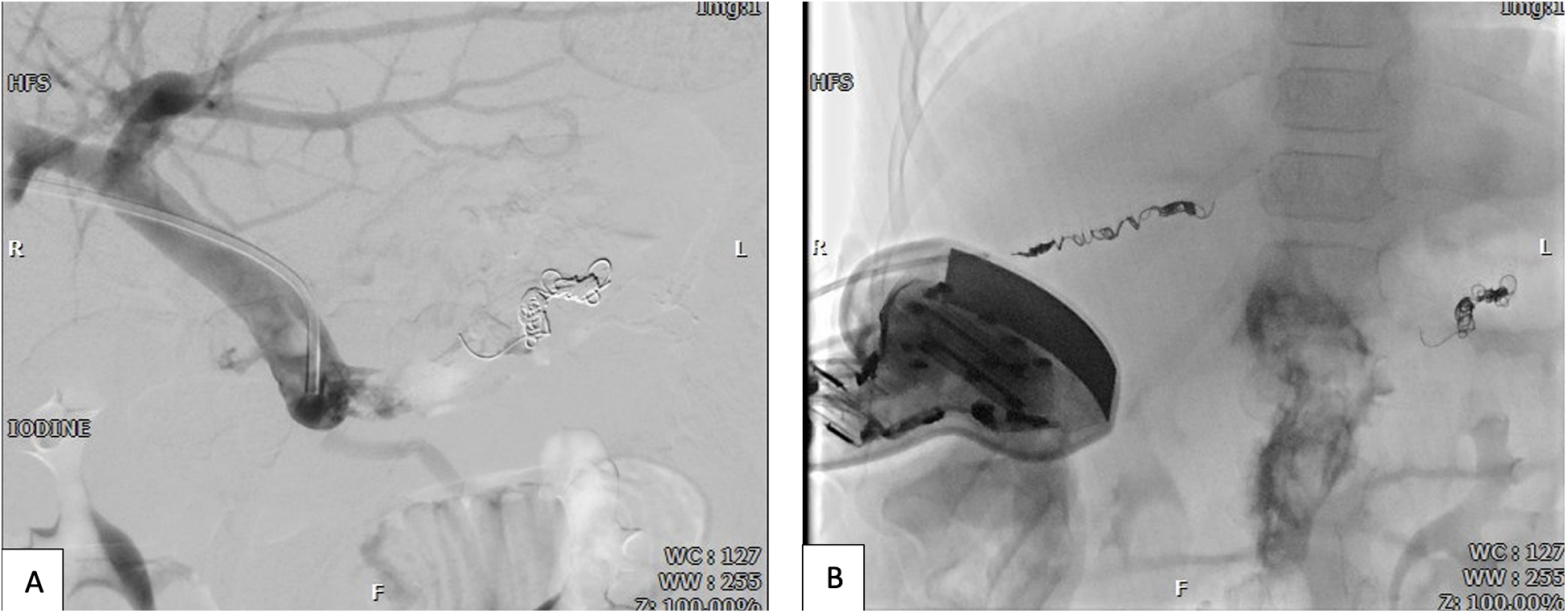
**(A)** Post-coiling and post-sclerotherapy trans-hepatic portography shows complete obliteration of varicose veins and porto-systemic shunts. **(B)** Hepatic sheath pathway coiling for hemostasis by guidance of sonography and fluoroscopy.

Regarding management, after conducting transhepatic portography (Figure 2A), intravenous sclerotherapy using the Coil-Assisted Retrograde Transvenous Obliteration (CARTO) method was applied. During the procedure, a 5F sheath was inserted transhepatically into the right portal vein to enable portography. Using a combination of Cobra and Vertebra catheters, portography revealed tortuous and dilated vessels associated with short gastric veins, which were connected to the inferior vena cava (IVC) and azygos vein. In order to manage the condition, a SCHELON microcatheter and AVIGO guidewire were used to access the proximal short gastric vein (Figure 3B). Sclerotherapy was performed with a mixture of 2 cc of sodium tetradecyl sulfate (SDS), 4 cc of air, and 1 cc of Lipiodol. Subsequently, two coils measuring 20 × 8 mm and 20 × 7 mm were deployed to achieve embolization (Figure 3). Portography post-procedure demonstrated no further entry of contrast into the dilated vessels (Figure 4A).

Given the minimal amount of SDS used, a follow-up CT angiography was performed and showed normal findings. The sheath in the portal vein was then removed, and a 16 × 40 mm coil was deployed in the transhepatic access tract to achieve hemostasis (Figure 4B). Post-procedure, hemoglobin levels returned to normal. To lower the risk of recurrence, secondary prophylaxis with beta-blockers was initiated.

## Discussion

Gastric variceal bleeding is a critical complication of portal hypertension, often presenting significant challenges in management due to its high morbidity and mortality rates. While conventional approaches such as Balloon-Occluded Retrograde Transvenous Obliteration (BRTO) (6) and Transjugular Intrahepatic Portosystemic Shunt (TIPS) (1) are well-established, they have limitations that necessitate alternative interventions in certain scenarios. CARTO and its modified variant (m-CARTO) have emerged as promising techniques, particularly in cases where complex anatomy or other contraindications preclude standard methods.

Two studies have evaluated role of CARTO in managing variceal bleeding. Lee et al. assessed CARTO in 20 patients with gastric variceal bleeding, reporting a 100% technical success rate. The patient cohort had a median age of 62 years, with most cases involving liver cirrhosis and a median Model for End-Stage Liver Disease (MELD) score of 10. CARTO achieved complete obliteration of varices and shunts, as confirmed by follow-up imaging. Importantly, there were no recurrences during the observation period. While the procedure was generally well-tolerated, one patient with severe comorbidities experienced systemic venous thrombosis, highlighting the need for careful patient selection (7). Furthermore, Uotani et al. demonstrated the efficacy of m-CARTO in a cohort of four patients with high-risk gastric varices (Hirota grade 4) and complex anatomical challenges. Complete thrombosis was achieved in all cases without recurrence during follow-up. However, one patient developed duodenal varices post-treatment, emphasizing the importance of long-term monitoring and addressing potential collateral complications (8).

In this case, CARTO method was chosen due to several reasons. While BARTO requires typically taking 4–5 h, CARTO takes considerably less time (9). Furthermore, BARTO requires a larger sheath, which posed a higher risk of bleeding in this case, as the transhepatic pathway was used. Notably, the transsplenic approach was not feasible since the patient had undergone a splenectomy. However, CARTO is not without limitations. CARTO is more technically demanding, often requiring the deployment of multiple detachable coils, which may increase the complexity and cost of the procedure. Despite these limitations, CARTO remains a valuable alternative, especially in high-risk patients with complex shunt anatomy, and we did not note any contraindications in this case. Notably, while in our case, balloon tamponade and endoscopic glue injection were not feasible due to logistical constraints, future studies should evaluate and compare the efficacy and outcomes of CARTO, BRTO, and endoscopic therapy in the pediatric population to establish the most effective treatment approach.

## Conclusion

CARTO and m-CARTO have demonstrated their efficacy in managing complex gastric varices, particularly in scenarios where BRTO and TIPS are contraindicated or fail. These methods provide a more targeted approach, avoiding systemic hemodynamic alterations associated with TIPS. Moreover, their ability to address complex anatomical challenges makes them particularly valuable in high-risk cases. Moving forward, more comparative studies are needed to refine patient selection criteria and optimize procedural techniques. Additionally, advancements in coil design and delivery systems could further reduce procedural times and enhance outcomes. The integration of imaging modalities and artificial intelligence could also play a role in improving procedural planning and predicting patient outcomes.

## Data Availability

The original contributions presented in the study are included in the article/Supplementary Material, further inquiries can be directed to the corresponding author.
