# Supplementary figures and images for: Coil-assisted retrograde transvenous obliteration for managing complex gastric variceal bleeding: a pediatric case report and review of techniques
Source: Front Pediatr. 2025 Apr 24;13:1558097. doi: 10.3389/fped.2025.1558097 (PMC12058803; doi:10.3389/fped.2025.1558097)

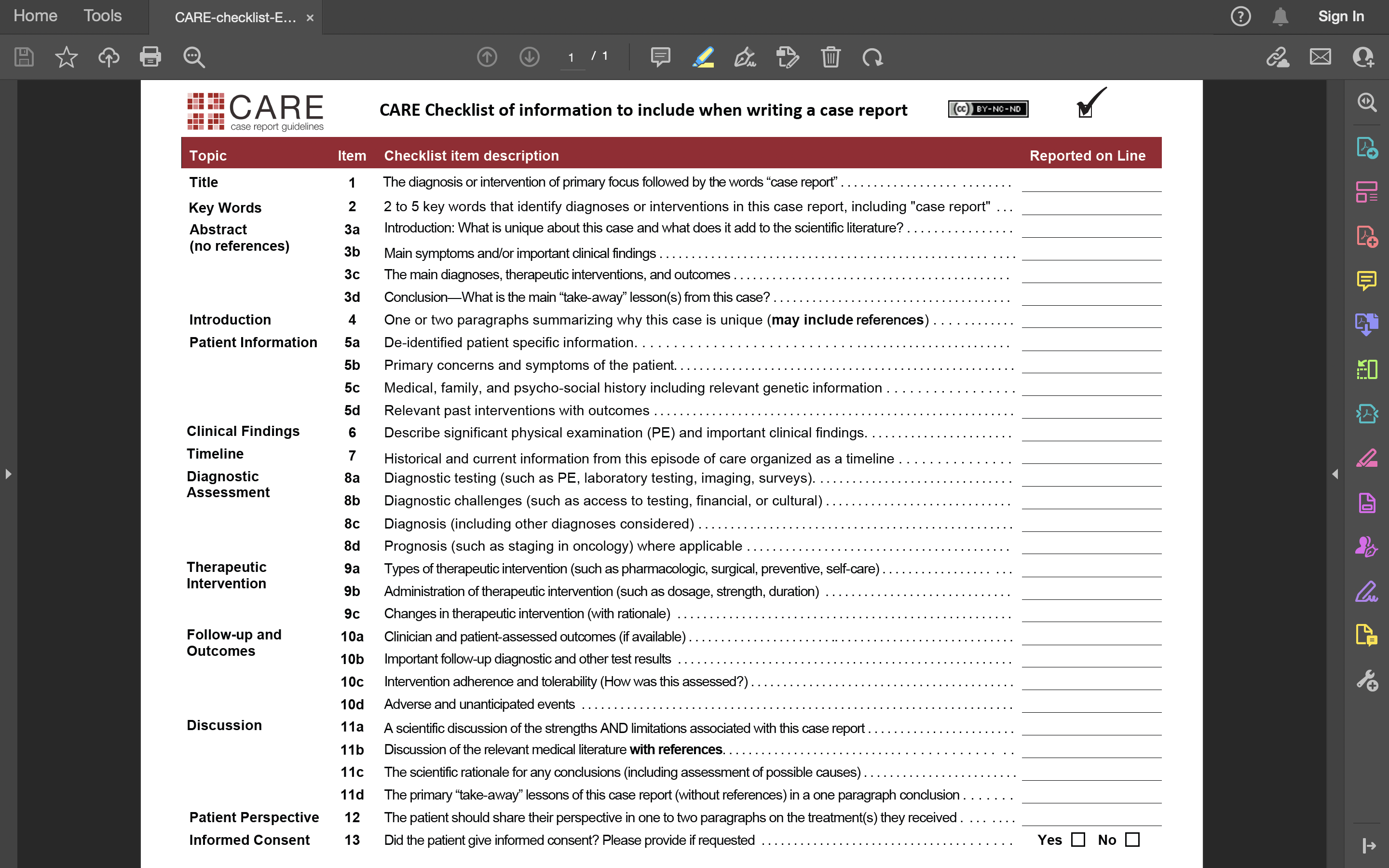


60-69

108

80-87

87-95

87-95

96-105

115-135

178-186

178-186

178-186

178-186

178-194

178-194

187-194

183-185

-

-

225-236; 281-292

-

-

286-292

286-292

286-292

286-292

360-392; 415-430

372-390

415-430

435-448

-

Supplement: Supplementary file 1 [file Supplementaryfile1.docx]
